# Maximising the Potential of Temporal $N_{e}$ Estimation in Long‐Term Population Monitoring Programmes

**DOI:** 10.1111/1755-0998.14125

**Published:** 2025-05-23

**Authors:** Tin‐Yu J. Hui

**Affiliations:** ^1^ Department of Life Sciences Imperial College London Ascot UK

**Keywords:** computer simulations, effective population size, genetic drift, genetic monitoring, temporal method

Effective population size ($N_{e}$) is indisputably one of the most important parameters in evolutionary biology. It governs the rate of evolution, magnitude of drift, effectiveness of selection, diversity, and many more. It also serves as a key indicator to inform population monitoring programmes, from conservation of endangered species to biocontrol of agricultural pests or disease vectors, and almost everything in between. In applications in which the contemporary $N_{e}$ is of interest, temporal F is one of the most widely used estimators. It measures the magnitude of drift among genetically neutral loci to estimate the harmonic mean $N_{e}$ between two time points. In this issue, Waples et al. (2025) present us a new software “MAXTEMP” to improve the precision of temporal F by incorporating additional samples outside of the focal period.

I was sceptical at first as these seemingly unrelated samples appear to be uninformative, especially after I had revisited Waples (2005) on the time periods at which the temporal $N_{e}$ estimates apply. Upon closer inspection, the authors reassured us with an intuitive yet robust argument: consider a population monitoring programme with initially two temporal samples sandwiching the focal period. A direct $N_{e}$ estimate is obtained via temporal F in the traditional way (Waples 1989). A third sample is subsequently collected, and the same $N_{e}$ can also be implied from the difference of the overall F and that between the second and third time points. There exists a weighted average (linear combination) of the two that outperforms either estimate by having a lower variance. The idea can be generalised to an arbitrary number of additional samples from which the implied estimates are calculated. The remaining challenge is to find an appropriate weighing system which the authors duly examined.

From a technical perspective, the direct and implied estimates share the same expectation hence the combined one remains largely unbiased. Without any additional cost (apart from sampling more individuals) MAXTEMP brings the benefit of variance reduction, whose magnitude depends on individual variances as well as their pairwise correlation. The implied estimates often come with much larger variances given they span across longer horizons. As all estimates aim to extract the same drift signal for the focal period positive correlations are unavoidably induced, limiting the potential to shrink the variance further. Despite these mathematical constraints the authors demonstrated a reduction in the standard deviation of F by up to 50% in selected cases. In my experience, the correlation weakens when the sample size s is limited, as sampling noise colludes with the underlying drift. This is hugely encouraging that the improvement is the greatest when it is most needed. While it is tempting to include as many additional samples as possible, the authors recommended to bring no more than one from either side or information will become saturated. It is also possible to couple temporal F with single‐sample estimates (e.g., Linkage Disequilibrium among unlinked loci) to resolve mystery $N_{e}$'s of unsampled generations.

In parallel with temporal F, the maximum likelihood (ML) approaches are being utilised to extract the same drift signal by considering the distributional change in allele frequency over time (Williamson and Slatkin 1999; Hui and Burt 2015). Both Waples et al. and I discovered that ML also benefits from having additional samples almost in the same way as the moment‐based F, with weights being automatically chosen during maximisation. This welcoming effect was not examined or documented in previous publications, although some suggested the possibility to aggregate multigenerational samples for non‐constant $N_{e}$ scenarios. Further investigation into the ML methods is required. Conceptually, MAXTEMP calculates F's from pairs of samples iteratively while ML jointly considers all data points, hence the idea of refining an earlier estimate does not exist in the latter. Given the lack of provision of software and the non‐trivial effort to alter ML algorithms to cater to individual sampling plans, the timely arrival of MAXTEMP fills in the current resource gap as a drop‐in alternative to existing tools (e.g., Do et al. 2014).

The latest development of temporal F has been shifting towards its application on genomic datasets, in which correlation among densely linked loci reduces the effective amount of information they hold (Hui et al. 2021; Waples et al. 2022). Even after accounting for pseudo‐replication sample size is likely to be the limiting factor for problems like this. The precision of F depends on $s/N_{e}$, meaning sample size cannot be perfectly determined *a priori*. Putting the issue of sample size aside, the ways in which MAXTEMP enhances $N_{e}$ estimation also depend on the true size. For small, fragmented populations a certain local $N_{e}$ has to be maintained to prevent inbreeding depression or mutational meltdown. Having tighter confidence intervals (C.I.) will undoubtably benefit the assessment of genetic health to ensure $N_{e}$ is always kept above a certain threshold. It also facilitates early detection of population decline as swift action is required to prevent any irreversible loss of genetic variation, or worse, extinction. On the other end of the spectrum where larger $N_{e}$ is of concern, the point estimate (or lower bound) of F can be negative which is interpreted as no drift with infinitely large $N_{e}$. Reducing the variance of F will lower the occurrence of yielding negative estimates as a direct consequence, or in other words, increase the chance of having meaningful $N_{e}$ and C.I. for decision making. It is foreseeable that with the aid of computer simulation the spirit of MAXTEMP will inspire future designs of population monitoring programmes, such as to incorporate samples from historic or pilot studies into the main analyses, or to develop contingency plans with post hoc sampling to rescue a negative $N_{e}$ estimate. With $N_{e}$ being recently incorporated as one of the headline indicators by the Convention on Biological Diversity (Thurfjell et al. 2022), the type of analyses requiring MAXTEMP is expected to grow. I am confident that the community will bring MAXTEMP to its full potential in years to come.

## Conflicts of Interest

The author declares no conflicts of interest.

## Data Availability

The author has nothing to report.
